# Diagnostic Performance of Three Serological Assays in Myasthenia Gravis: A Prospective Multicentre Study

**DOI:** 10.1111/sji.70072

**Published:** 2025-11-24

**Authors:** Laura Cuomo, Laura De Giglio, Maria Antonietta Isgrò, Maria Totaro, Matteo Pratali, Caterina Fragomeli, Lorenzo Cosmi, Francesco Annunziato, Boaz Palterer, Maria Concetta Altavista, Giovanni Antonini, Marianna Brienza, Chiara Cambieri, Francesca Cortese, Laura Fionda, Francesca Gragnani, Maurizio Inghilleri, Stefania Morino, Antonio Petrucci, Carlo Piantadosi, Eleni Anastasiadou, Marina Vitillo, Pankaj Trivedi, Elena Maria Pennisi

**Affiliations:** ^1^ U.O.C. Clinical Pathology San Filippo Neri Hospital, ASLRoma1 Rome Italy; ^2^ Neuromuscular and Rare Neurological Diseases Center, Neurology Unit San Filippo Neri Hospital, ASLRoma1 Rome Italy; ^3^ SOD Immunoallergology AOU Careggi Florence Italy; ^4^ Department of Experimental Medicine and Clinic Università of Florence Florence Italy; ^5^ NESMOS Department Sapienza University of Rome and UniCamillus‐St. Camillus International University of Health Science Rome Italy; ^6^ Department of Human Neurosciences Sapienza University of Rome Rome Italy; ^7^ Neuromuscular Disease Center Sant'andrea Hospital Rome Italy; ^8^ Neurology Unit Ospedale Pertini Rome Italy; ^9^ Center for Neuromuscular and Neurological Rare Disease San Camillo Forlanini Hospital Rome Italy; ^10^ Neurology Unit Ospedale San Giovanni Rome Italy; ^11^ Department of Clinical and Molecular Medicine Sapienza University Rome Italy; ^12^ Department of Experimental Medicine Sapienza University Rome Italy

**Keywords:** AChR, ELISA, F‐CBA, MuSK, myasthenia gravis, RIPA

## Abstract

Anti‐acetylcholine receptor (AChR) and anti‐muscle‐specific tyrosine kinase (MuSK) autoantibody detection is crucial in the diagnosis and choice of treatment of myasthenia gravis (MG). We conducted a multicentre prospective study comparing radioimmunoprecipitation assay (RIPA), enzyme‐linked immunosorbent assay (ELISA) and fixed cell‐based assay (F‐CBA) for anti‐AChR and anti‐MuSK antibody detection in 78 patients with suspected MG with at least 6 months of clinical follow‐up. In the diagnosis of seropositive MG (anti‐AChR+anti‐MuSK abs), RIPA was most sensitive (82.8%) compared to ELISA (81.0%) and F‐CBA (70.7%). F‐CBA exhibited the highest specificity overall (95.0%). For anti‐AChR detection, F‐CBA demonstrated a sensitivity of 73.6% and specificity of 95.0%; ELISA showed sensitivity and specificity of 81.1% and 85.0%, respectively; and RIPA yielded sensitivity and specificity of 81.1% and 95.0%. Sensitivity of F‐CBA improved when sera were tested at lower dilution (1:5) versus the manufacturer's recommended 1:10, without compromising specificity. Agreement among methods was almost perfect for anti‐AChR detection (Cohen's Kappa > 0.81). For anti‐MuSK detection, agreement was substantial between ELISA and RIPA, moderate between ELISA and F‐CBA, and fair between RIPA and F‐CBA. Higher anti‐AChR antibody levels were found in generalised versus ocular MG by both RIPA and ELISA. F‐CBA confirmed its optimal specificity while the sensitivity seems to be influenced by sample dilution. In conclusion, given the radioactive nature of RIPA and consequent limitations, F‐CBA may represent a valid alternative in anti‐AChR and anti‐MuSK antibody detection in MG diagnosis. We suggest that the use of live‐CBA or RIPA could be reserved for inconclusive cases.

## Graphical abstract

A comparison of three serological methods for the diagnosis of myasthenia gravis.

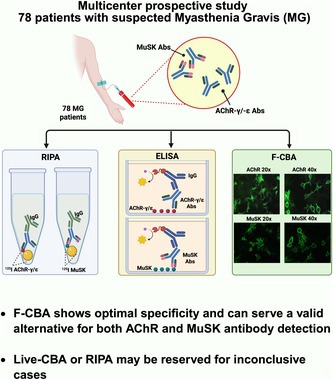

## 1 Introduction

Myasthenia gravis (MG) is an autoimmune disorder characterised by impairment of neuromuscular junction mediated by antibodies directed mainly against the acetylcholine receptor (AChR). Presence of anti‐AChR antibodies accounts for approximately 50% of ocular and 85% of generalised MG, while an additional 5%–10% MG patients are positive for antibodies against muscle‐specific kinase (MuSK) and low‐density lipoprotein receptor‐related protein 4 (LRP4). A proportion of MG patients (~10%) are seronegative (SN‐MG) for antibodies [1]. Determination of antibody status is therefore important for a definitive diagnosis and can orient to specific immune treatments.

The currently utilised methods differ from an economic and feasibility point of view. Radioimmunoprecipitation assay (RIPA) is presently considered the gold standard method, but the disadvantage of employing radioactive isotopes is making it progressively limited [2]. Enzyme‐linked immunosorbent assay (ELISA) is widely used in many laboratories. Based on direct or competitive methods, different ELISA assays have been commercialised but results on their accuracy are discordant [3]. Live‐cell‐based assay (L‐CBA) is utilised only in selected centres because it requires laboratory equipment for cell cultures and specialised staff [4]. In the last few years, a mosaic biochip, the fixed‐cell‐based assay (F‐CBA) is commercially available. The principle of the method is based on the detection of fetal (AChR‐γ) and adult (AChR‐ε) subunits of AChR and MuSK, transfected in human embryonic kidney (HEK) cells together with the clustering protein rapsyn, by indirect immunofluorescence (IIF). Ever since its commercialisation, some studies have compared CBA with the other methods. A majority of these studies were retrospective and compared CBA with another method [2, 5, 6, 7].

Here we carried out a prospective study conducted on suspected MG patients recruited from six local neuromuscular disease centres in Rome, in whom the myasthenia diagnosis was evaluated both neurophysiologically and by the response to treatment after a clinical follow‐up of 6 months.

We evaluated the diagnostic performances of F‐CBA, RIPA and ELISA for anti‐AChR alone and combined with anti‐MuSK antibodies in MG diagnosis, the level of agreement among methods and the comparison of RIPA and ELISA anti‐AChR antibody concentration in generalised (GMG) and ocular (OMG) variants of MG.

## 2 Material and Methods

### 2.1 Population

**FIGURE 1:**
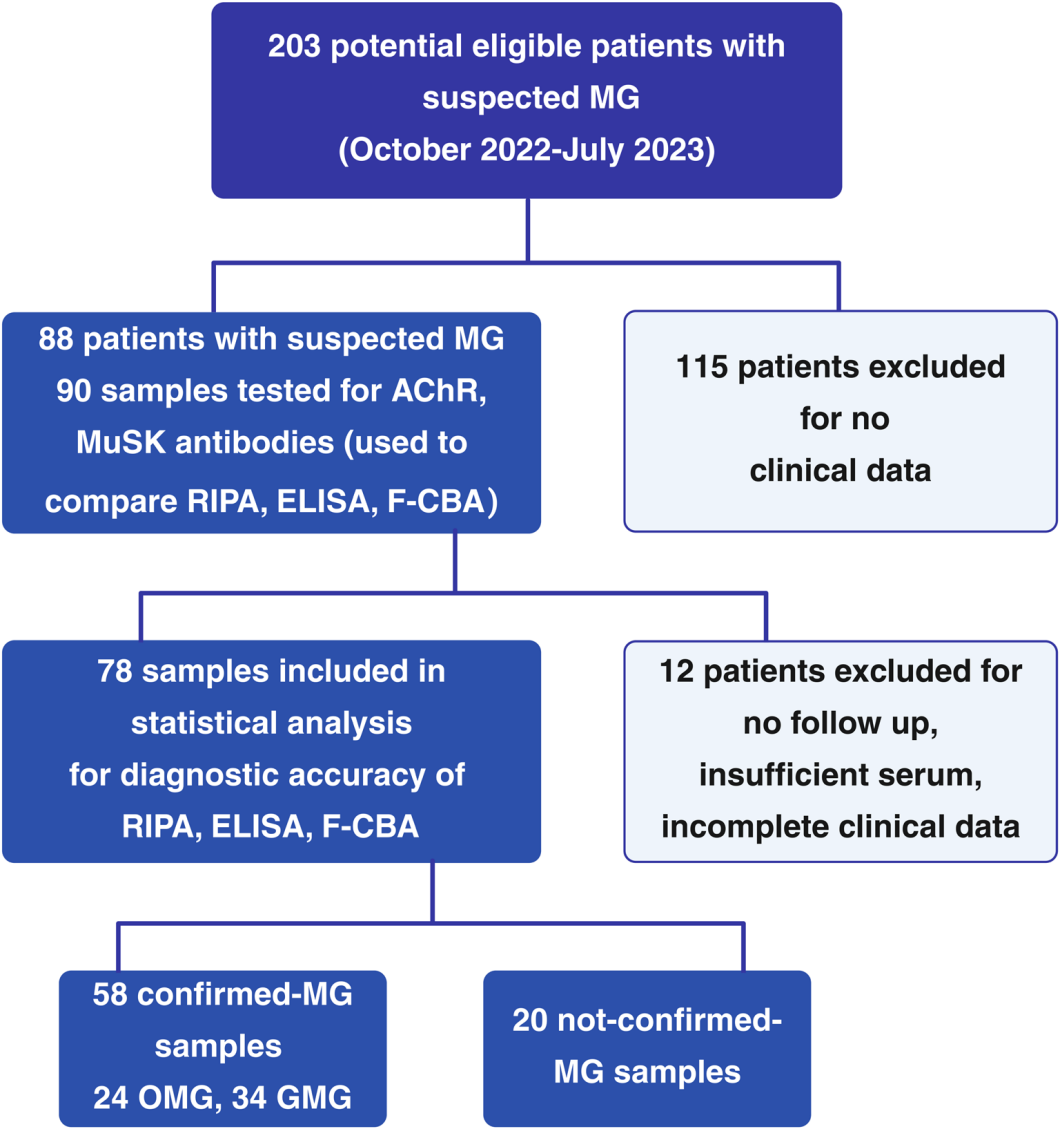
Flow diagram of patient recruitment. Out of 203 patients referred to six neuromuscular centres in Rome with medical request for anti‐AChR and anti‐MuSK antibodies, 88 patients (90 samples) were eligible according to criteria described in the Material and Methods section. These patients were tested for anti‐AChR and anti‐MuSK antibodies with the three methods. The data were used to estimate agreement and Cohen's kappa. Three samples of the same patient who underwent follow up were included in the statistical analysis. Out of 88 patients, 78 with complete clinical evaluation were enrolled for statistical analysis to determine diagnostic accuracy of the three methods.

Between October 2022 and June 2023, 203 patients from six neuromuscular centres in Rome were referred to our laboratory for anti‐AChR and anti‐MuSK abs dosage for the first stage of a diagnostic workup for suspected MG (Figure 1). Out of this group, 88 patients (mean age 62 ± 16 years, 43 males and 45 females) were tested for anti‐AChR and anti‐MuSK antibodies detection with RIPA, ELISA and F‐CBA to compare method concordance. From this group, 12 patients were further excluded due to uncertain diagnosis, incomplete clinical data and/or insufficient serum samples. Thus the remaining 78 eligible participants included 58 confirmed MG (24 OMG, 34 GMG) and 20 non‐confirmed MG patients (Figure 1). MG was confirmed in each patient by the Neuromuscular centre of reference, according to Italian recommendation guidelines [8]. All the subjects were divided into those with or without MG based on the presence of clinical symptoms (ptosis, diplopia, dysphagia, muscular fatigability, etc.), repetitive nerve stimulation, single fiber study and response to MG therapy (pyridostigmine and immunosuppressive drugs). When the diagnosis was doubtful, patients were re‐evaluated after a period of follow‐up of 2–6 months to retest treatment response, neurophysiological behavior and diagnosis assignment. The study was conducted according to the Helsinki declaration and informed consent was obtained from all patients.

### 2.2 Anti‐AChR and Anti‐MuSK Antibodies Detection

Three aliquots of serum sample from each patient were collected and stored at −20°C until analysis. Two aliquots were separately processed for ELISA and F‐CBA testing. The third aliquot was renumbered and sent unidentified in dry ice to the Department of Experimental and Clinical Medicine, University of Florence for RIPA antibodies determination. Anti‐AChR and anti‐MuSK antibody determination by RIPA was performed using commercial kits (RSR distributed by PANTEC) according to the manufacturer's instructions. The following formula was used to calculate abs concentration:

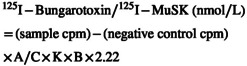

where A represents radioactivity on the day of the assay, C is the sample volume (μL); K is the specific acitivity of ^125^I (Ci/mmol), B is the *y*‐counter efficiency.

ELISA kits for anti‐AChR (Euroimmun, Lubeck, Germany) and for anti‐MuSK antibodies determination (IBL, International GmbH, Hamburg, Germany), were used according to the manufacturer's instructions and performed on the Analyser I instrument (Euroimmun). F‐CBA was assessed using the Mosaic‐2 Biochip from Euroimmun which simultaneously detects antibodies against fetal (AChR‐γ) or adult (AChR‐ε) forms of AChR and MuSK. An additional well containing wild‐type control EU‐90 cells was also present as an internal negative control. Sera were considered positive when two expert operators, independently detected a positive reaction at the dilution of 1:10. Additional serum dilution (1:5) was tested in selected samples. Slides were prepared manually according to the manufacturer's instructions and analysed under a Nikon fluorescence microscope (Nikon Eclipse).

### 2.3 Statistical Analysis

Statistical analysis was performed using Statistical Package for the Social Sciences (SPSS) version 27.0. Sensitivities, specificities, and overall diagnostic accuracies of the three methods were evaluated on 78 participants with complete clinical data. Receiver operating characteristic (ROC) curves for RIPA, ELISA, and F‐CBA were evaluated, and the corresponding areas under the curve (AUC) were calculated exclusively for anti‐AChR antibodies, due to the small number of patients positive for anti‐MuSK antibodies; thus, MG patients positive for anti‐MuSK antibodies were excluded. The Mann–Whitney *U* test was used to compare median anti‐AChR antibody levels between OMG and GMG patients. Agreement between methods was calculated using results from 90 samples with MedCalc software. Cohen's kappa was employed to measure agreement between methods: according to the literature, kappa values of 0 < *κ* ≤ 0.20 indicate poor agreement; 0.21 < *κ* ≤ 0.40, fair agreement; 0.41 < *κ* ≤ 0.60, moderate agreement; 0.61 < *κ* ≤ 0.80, good agreement; and 0.81 < *κ* ≤ 1.00, very good agreement [9]. The positive and negative predictive values were calculated by the contingency table.

## 3 Results

### 3.1 Diagnostic Performance Analysis of the Three Methods: RIPA, ELISA and F‐CBA

As reported in Table 1, RIPA and ELISA showed similar performance in detecting autoantibodies (anti‐AChR and anti‐MuSK) in 48/58 and 47/58 MG patients, respectively, while F‐CBA used at 1:10 serum dilution, turned positive in 41/58 MG patients. RIPA, ELISA, and F‐CBA detected autoantibodies more effectively in GMG patients (88.2%, 91.1%, and 82.3%, respectively) than in OMG patients (75.0%, 70.8%, and 54.1%, respectively).

**TABLE 1:** Anti‐AChR and anti‐MuSK antibodies detection in 78 enrolled patients with the three methods.

Method	MG	GMG	OMG	NOMG
AChR + MuSK^a^	*n* = 58^b^	*n* = 34	*n* = 24	*n* = 20
RIPA				
Positive	82.7% (48/58)	88.2% (30/34)	75.0% (18/24)	10.0% (2/20)
Negative	17.2% (10/58)	11.7% (4/34)	25.0% (6/24)	90.0% (18/20)
ELISA				
Positive	81.0% (47/58)	91.1% (31/34)	70.8% (17/24)	15.0% (3/20)
Negative	18.9% (11/58)	8.8% (3/34)	29.1% (7/24)	85.0% (17/20)
F‐CBA (1:10)^c^				
Positive	70.6% (41/58)	82.3% (28/34)	54.1% (13/24)	5.0% (1/20)
Negative	29.3% (17/58)	17.6% (6/34)	45.8% (11/24)	95.0% (19/20)
F‐CBA (1:5)				
Positive	75.8% (44/58)	82.3% (28/34)	66.6% (16/24)	5.0% (1/20)
Negative	24.1% (14/58)	17.6% (6/34)	33.3% (8/24)	95.0% (19/20)

Abbreviations: GMG, generalised myasthenia gravis; MG, myasthenia gravis; *n*, number of tested subjects; NOMG (patients initially suspected for MG where diagnosis was not confirmed); OMG, ocular myasthenia gravis.

^a^ Combined AChR ab and MuSK ab indicate that both antibody types were concurrently analysed to assess the diagnostic performance of CBA, RIPA and ELISA.

^b^ Out of 58 MG cases, four (three GMG and one OMG) were affected with thymoma.

^c^ Dilution recommended by the manufacturer.

In 20 patients where MG diagnosis was not confirmed after follow‐up (NOMG), F‐CBA detected only one positive sample (a patient affected by amyotrophic lateral sclerosis [ALS]), accounting for 5% of NOMG patients. Meanwhile, RIPA detected autoantibodies in two NOMG (10%), and ELISA in three NOMG patients (15%).

The highest overall diagnostic accuracy in MG diagnosis (combined anti‐AChR and anti‐MuSK detection) was found for RIPA (84.6%), followed by ELISA (82.1%) and F‐CBA (76.9%) (Table 2A). RIPA displayed higher sensitivity (82.8%) than ELISA (81.0%) and F‐CBA 1:10 (70.7%), while F‐CBA provided the highest specificity (95.0%) compared to RIPA (90.0%) and ELISA (85.0%). In addition, 26 ANA and eight positive sera were found negative for anti‐AChR and anti‐MuSK antibodies by F‐CBA, thus confirming the method's specificity (not shown).

**TABLE 2:** Diagnostic performance of RIPA, ELISA, F‐CBA in MG diagnosis (A) and in anti‐AChR (B) antibodies detection.

AChR + MuSK	RIPA	ELISA	F‐CBA (1:10)	F‐CBA (1:5)
**(A)**				
SE (95% CI)	82.8 (73.0–92.5)	81.0 (70.9–91.1)	70.7 (59.0–82.4)	75.9 (64.8–86.9)
SP (95% CI)	90.0 (76.9–100.0)	85.0 (69.4–100.0)	95.0 (85.4–100.0)	95.0 (85.4–100)
PPV %	96.0	94.0	97.6	97.8
NPV %	64.3	60.7	52.8	57.6
LR+	8.2	5.4	14.1	15.1
LR−	0.19	0.22	0.30	0.25
DOR	43.1	24.2	45.8	59.7
Diagnostic accuracy %	84.6	82.1	76.9	80.8

AChR	RIPA	ELISA	F‐CBA (1:10)	F‐CBA (1:5)
**(B)**				
SE (95% CI)	81.1 (70.6–91.7)	81.1 (70.6–91.7)	73.6 (61.7–85.5)	79.2 (68.3–90.2)
SP (95% CI)	95.0 (85.4–100.0)	85.0 (69.4–100.0)	95.0 (85.4–100.0)	95.2 (85.4–100.0)
PPV %	97.7	93.5	97.5	97.7
NPV %	65.5	63.0	57.6	63.3
LR+	16.2	5.4	14.7	15.8
LR−	0.19	0.22	0.27	0.21
DOR	81.5	24.4	52.9	72.7
Diagnostic accuracy %	84.9	82.2	79.5	83.6

*Note:* The recommended cut‐off value of RIPA, ELISA and F‐CBA for anti‐AChR antibodies was 0.50, 0.40 nmol/L and titre 1:10 respectively; for anti‐MuSK antibodies was 0.05 nmol/L, 0.40 U/mL and titre 1:10 respectively. F‐CBA was additionally tested at 1:5 sample dilution.

Abbreviations: DOR, diagnostic odds ratio; LR, likelihood ratio; NPV, negative predictive value; PPV, positive predictive value; SE, sensitivity; SP, specificity.

Fixed cell‐based assay (F‐CBA) was further performed at a lower serum dilution (1:5). As shown in Tables 1 and 2, using a 1:5 serum dilution allowed the detection of anti‐AChR antibodies in three additional OMG patients, increasing F‐CBA assay sensitivity to 75.9% without affecting its specificity (95.0%).

Table 2 reports the diagnostic odds ratio (DOR) of each method along with related measures of diagnostic accuracy, including sensitivity (SE), specificity (SP), positive predictive value (PPV), negative predictive value (NPV), positive likelihood ratio (LR+) and negative likelihood ratio (LR−). A very high DOR was found when considering methods in MG diagnosis, for F‐CBA 1:10/1:5 (45.8/59.7) and RIPA (43.1), compared to ELISA (24.2).

### 3.2 Diagnostic Accuracy Analysis for Anti‐AChR Antibodies With the ROC Curve

**FIGURE 2:**
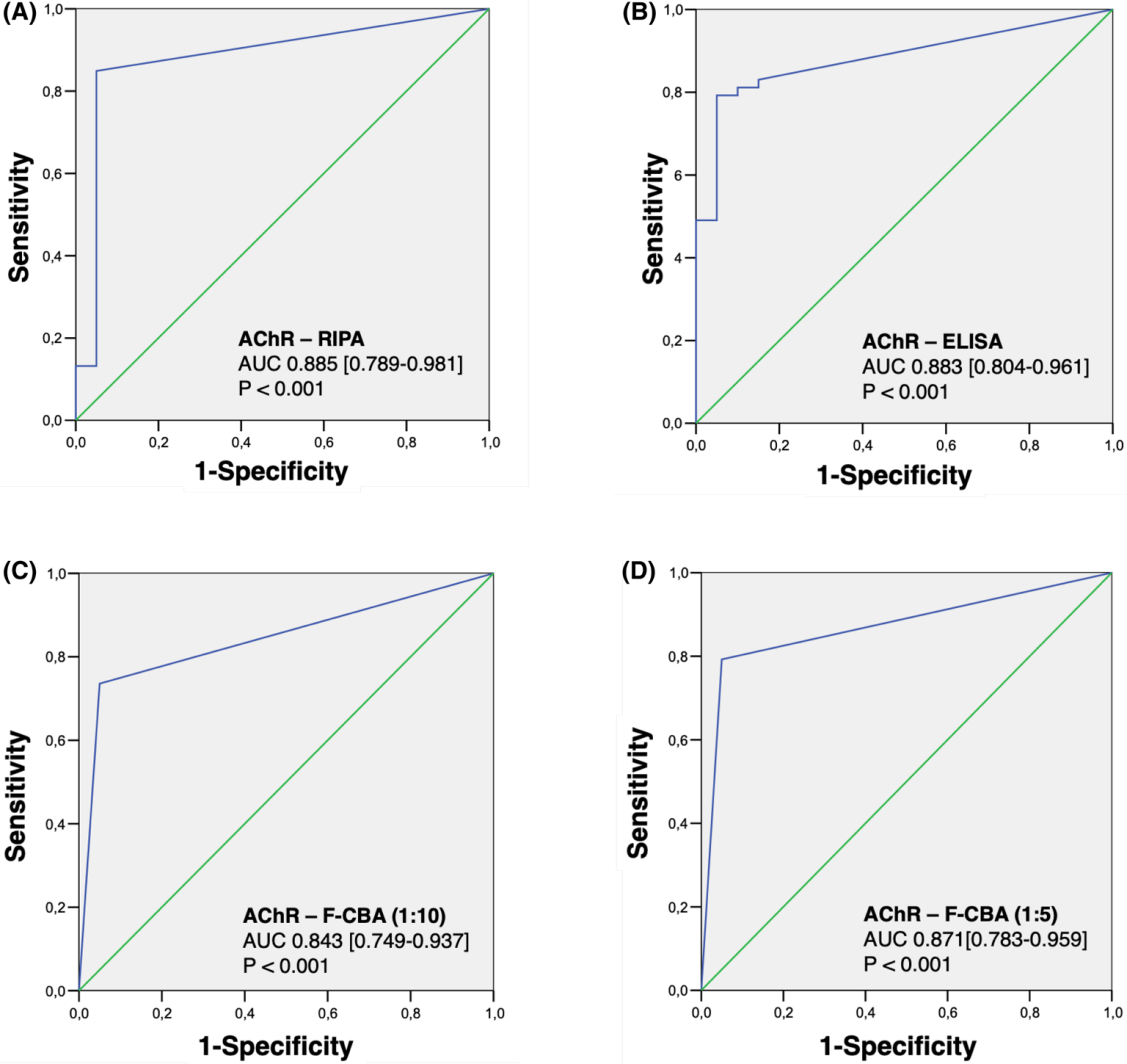
The receiver operating characteristic (ROC) curve of AChR antibodies detected by RIPA, ELISA and F‐CBA. The area under the ROC curve (AUC 0–1) reflects the diagnostic accuracy of RIPA (A), ELISA (B), F‐CBA 1:10 (C) and 1:5 (D) for AChR antibodies, with values closer to 1 indicating higher accuracy. Statistical analysis was performed by using SPSS version 27.0. Patients positive for anti‐MuSK antibodies were excluded from this analysis.

As shown in Figure 2, the AUC value of RIPA (0.885, 95% CI: 0.789–0.981, *p* < 0.001) was higher than that of ELISA (AUC = 0.883, 95% CI: 0.804–0.961, *p* < 0.001) and F‐CBA (1:10 AUC = 0.843, 95% CI: 0.749–0.937, *p* < 0.001; 1:5 AUC = 0.871, 95% CI: 0.783–0.959, *p* < 0.001). Figure 2 and Table 2B also present the related diagnostic accuracy parameters for each method.

### 3.3 Comparison of Anti‐AChR Antibodies Levels in OMG and GMG

**FIGURE 3:**
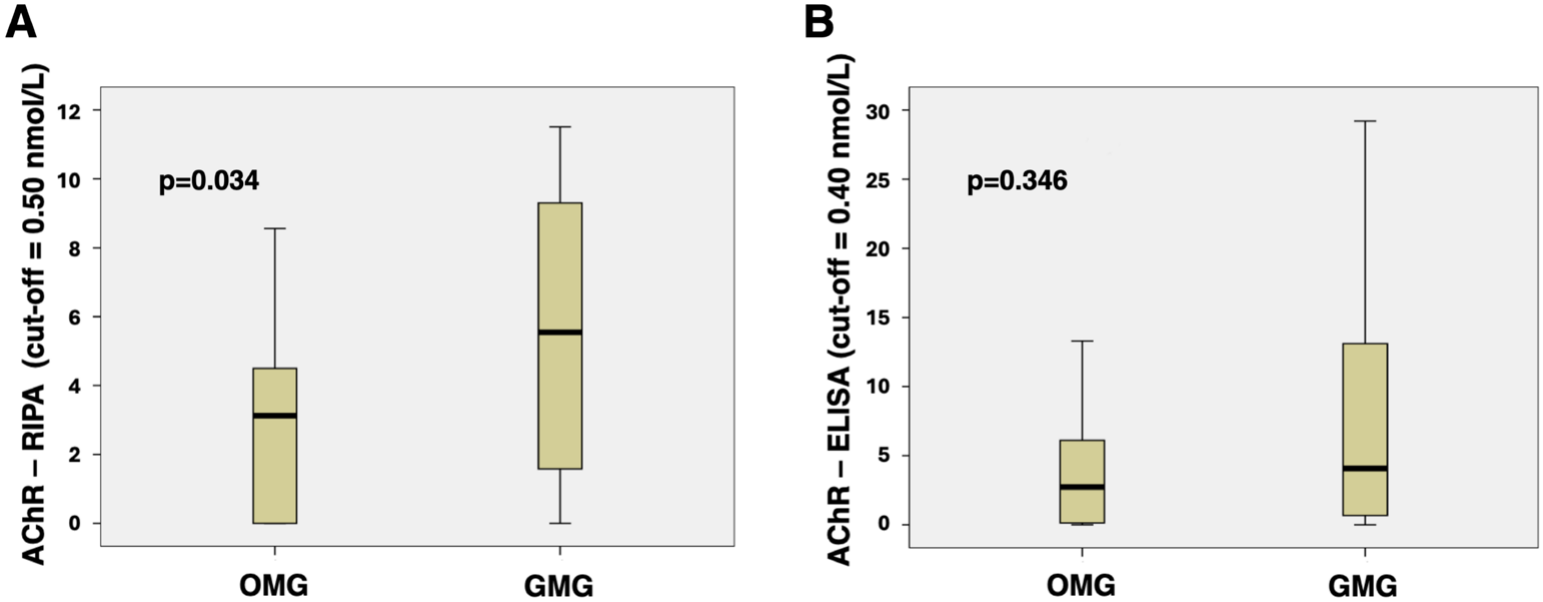
Comparison of anti‐AChR antibodies levels. By RIPA (A) and ELISA (B) in OMG (*n* = 24) and GMG (*n* = 34). Statistical analysis was performed using Mann–Whitney Test.

Using the RIPA method, OMG patients showed significantly lower anti‐AChR antibody levels than GMG patients (median 3.13 nmol/L versus 5.55 nmol/L, *p* = 0.034) (Figure 3A). The ELISA method revealed median anti‐AChR antibody levels of 2.73 nmol/L in OMG patients compared to 4.08 nmol/L in GMG patients (*p* = 0.346, not significant) (Figure 3B).

### 3.4 Agreement Between RIPA, ELISA and F‐CBA Assays for Anti‐AChR Antibodies Detection

The level of agreement among methods was evaluated based on the results of anti‐AChR and anti‐MuSK antibody detection in 90 samples. As shown in Table 3, the agreement percentages for ELISA versus RIPA, ELISA versus CBA, and RIPA versus F‐CBA were 96.6%, 92.2%, and 95.5%, respectively, for anti‐AChR antibodies, and 94.4%, 96.6%, and 91.1%, respectively, for anti‐MuSK antibody detection. The Cohen's kappa coefficient for anti‐AChR antibody detection indicated very good agreement for ELISA/RIPA (0.93), ELISA/F‐CBA (0.84), and RIPA/CBA (0.91). For anti‐MuSK antibody detection, the Cohen's kappa coefficient indicated good agreement for ELISA/RIPA (0.63), moderate agreement for ELISA/CBA (0.55), and fair agreement for RIPA/CBA (0.30).

**TABLE 3:** Concordance of assays for anti‐AChR and anti‐MuSK antibodies detection.

Method	Cohen's Kappa (95% CI)	Agreement (%)	+/+^b^	+/−	−/+	−/−
AChR						
ELISA vs. RIPA	0.93 (0.85–1.00)	96.6	45.0	3.0	0.0	42.0
ELISA vs. F‐CBA						
1:10	0.84 (0.73–0.95)	92.2	41.0	7.0	0.0	42.0
1:5	0.91 (0.827–0.99)	95.5	44.0	4.0	0.0	42.0
RIPA vs. F‐CBA						
1:10	0.91 (0.82–0.99)	95.5	41.0	4.0	0.0	45.0
1:5	0.97 (0.93–1.00)	98.9	44.0	1.0	0.0	45.0
MuSK						
ELISA vs. RIPA	0.63 (0.35–0.92)	94.4	5.0	0.0	5.0	80.0
ELISA vs. F‐CBA^a^	0.55 (0.11–0.99)	96.6	2.0	3.0	0.0	85.0
RIPA vs. F‐CBA^a^	0.30 (0.02–0.63)	91.1	2.0	8.0	0.0	80.0

^a^ F‐CBA was tested at 1:10 dilution only.

^b^ +/+, number of cases positive with both methods; +/−, number of cases positive by the first method but negative by the second, etc.

## 4 Discussion

During recent years, several studies have examined the diagnostic accuracy of L‐CBA and, more recently, the newly commercialised F‐CBA, using RIPA and ELISA as reference methods [3, 5, 6, 10, 11]. These comparative studies have demonstrated excellent specificity and higher sensitivity of both live and fixed CBA compared to RIPA in detecting anti‐AChR and anti‐MuSK antibodies in patients considered seronegative by other methods. This improved sensitivity is likely related to the recognition of high receptor density in transfected cells and the recruitment of low‐affinity anti‐AChR antibodies. However, these studies were retrospective; participant selection was primarily based on autoantibody detection by RIPA, and the diagnostic performance in MG diagnosis was evaluated separately for anti‐AChR and anti‐MuSK antibodies.

In the present multicentre prospective study, anti‐AChR and anti‐MuSK antibodies detection with RIPA, ELISA and F‐CBA was carried out in a single sample in each patient. We enrolled patients only if their complete clinical information and follow‐up was available. As an adequate control group, we selected patients with a clinical suspicion and aimed to understand and assess the diagnostic performance of the test. Neurophysiological and clinical data in supporting MG diagnosis were evaluated after obtaining serological results. We found that RIPA could identify 82.7% MG‐affected patients by anti‐AChR and anti‐MuSK combined antibodies detection. Regarding the specificity, two NOMG patients were found autoantibodies positive by RIPA, one of whom was affected by ALS and the other was borderline for anti‐MuSK antibodies (0.07 nmol/L, cut‐off = 0.05 nmol/L). It has been reported that levels of anti‐AChR antibodies could be high in ALS patients, but its significance remains unknown [12]. Indeed, the same ALS patient in our study was the only NOMG patient who was anti‐AChR positive also by the F‐CBA method. Similar results were obtained by ELISA, which could detect anti‐AChR and anti‐MuSK antibodies in 81% MG patients, with the lowest specificity (85%) compared to RIPA and F‐CBA. ELISA was more effective in autoantibodies identification in GMG compared to RIPA, while RIPA was the best performing method in autoantibodies detection in OMG patients.

In our study, F‐CBA was the most specific method; however, it was the least sensitive, likely due to the fixation process, which may impair antigen–antibody binding, thus reducing antibody binding. Consequently, F‐CBA failed at standard dilution to identify four anti‐AChR positive samples that were detected by both other methods. Clinical evaluation indicated that these four individuals were OMG patients with a statistically significant lower median level of anti‐AChR antibodies when measured by RIPA (Figure 3). Notably, lower antibody levels have been reported in OMG [13]. We observed that diluting the samples at 1:5 increased the sensitivity of F‐CBA (SE 75.8%), resulting in three out of the four previously mentioned samples testing positive, without compromising specificity. The remaining negative specimens were unaffected when tested at the lower dilution.

Presently, only two prospective studies have been conducted, based on Chinese and Brazilian cohorts, respectively [14, 15]. In both investigations, F‐CBA outperformed ELISA in terms of sensitivity and specificity. Li et al. [14] reported F‐CBA sensitivity and specificity values for anti‐AChR (and combined anti‐AChR and anti‐MuSK detection) in agreement with our study, notwithstanding a different CBA brand. In contrast, sensitivity values for RIPA and ELISA were significantly lower compared to our findings and those commonly reported in the literature [16]. The weak performance of RIPA and ELISA in that study [14] may apparently make F‐CBA as having a higher diagnostic accuracy. The South American study [15] found F‐CBA to have comparable diagnostic accuracy to RIPA for anti‐AChR antibody detection. However, the study did not evaluate combined anti‐AChR and anti‐MuSK performance and excluded borderline RIPA values.

In our study F‐CBA and ELISA assays displayed an optimal agreement for anti‐AChR antibodies detection compared to the gold standard RIPA test (Cohen's *k* = 0.93 and 0.91 respectively). Agreement was quite fair when anti‐MuSK antibodies detection was considered probably due to the low number of positive cases. A major disadvantage of RIPA is the use of radioactive isotopes. Due to this, clinical laboratories have significantly reduced its use. Hence, ELISA and F‐CBA may be used to avoid radioactive handling without dramatically diminished sensitivity for anti‐AChR and anti‐MuSK antibodies detection in MG diagnosis. Consistent with our data, a recent study found that F‐CBA could even improve MG diagnostic accuracy compared to RIPA, in atypical clinical presentations of the disease [17]. A lower sample dilution than the one suggested by the manufacturer, could be employed to improve the sensitivity of the F‐CBA method. However, studies with larger cohorts of patients will be required to confirm its validity.

In conclusion, based on our data we suggest that F‐CBA could be used in lieu of RIPA to avoid radioactive handling for MG diagnosis. In cases of doubtful negative results, L‐CBA, currently conducted by select research laboratories with cell culture facilities, and RIPA could be performed as second‐level assays by reference laboratories.

## Author Contributions

L.C., M.V., E.M.P. conceived the study. L.C., M.T., M.P., C.F. performed experiments. L.D.G., M.A.I., E.A. performed statistical analysis. L.D.G., G.A., M.B., C.C., F.C., L.F., F.G., M.I., S.M., A.P., M.C.A., C.P., E.M.P. followed patients and collected clinical data. L.Co., F.A., B.P., P.T., E.M.P. supervised the study. L.C., P.T. and E.M.P. wrote the paper.

## Funding

This work was supported by LILT (5x1002021), Ministero dell'Università e della Ricerca (PRIN2022).

## Conflicts of Interest

The authors declare no conflicts of interest.

## Data Availability

The data that support the findings of this study are available from the corresponding author upon reasonable request.
